# A catena between psychiatric disorders and non‐scarring alopecias—A systematic review

**DOI:** 10.1002/ski2.194

**Published:** 2022-11-25

**Authors:** Ana L. Forneris Crego, Anastasia Therianou, Parastoo Hashemi, Claire A. Higgins

**Affiliations:** ^1^ National Heart and Lung Institute Imperial College London London UK; ^2^ Department of Dermatology Imperial College NHS Healthcare Trust London UK; ^3^ Department of Bioengineering Imperial College London London UK

## Abstract

For many years, clinical observations have suggested that there is an intrinsic connection between psychological state and skin diseases. Stress responses are typically mediated by several hormones, which are modulated *via* the hypothalamic‐pituitary‐adrenal axis. This typical stress response is not only one theory for psychiatry disorder pathophysiology, but it also modifies hair growth by altering the skin's inflammatory environment. Given that different forms of hair loss, such as androgenetic alopecia, alopecia areata, or telogen effluvium, and hair follicle cycling can be altered by immune cells within the follicle milieu, we hypothesized that specific forms of hair loss are correlated to psychiatric illnesses. To address this, we conducted a systematic review by searches in April and May 2021 through Ovid MEDLINE and PUBMED (ranging from 1951 to the present day), identifying 179 reports. A further 24 reports were identified through website and citation searches giving a total of 201 reports. After applying exclusion criteria, 21 papers were reviewed, and 17 were included for data analysis. It is undeniable that hair loss greatly affects Health‐related Quality of Life (HrQol) and it is heavily associated with major depressive disorder and anxiety. The correlation between hair loss and mental health disorders was significant, however, due to the low number of publications with quantitative data we were not able to identify correlations between each hair loss type with each psychiatric disorder. Further studies to better connect specific hair loss diseases to specific disorders are therefore critical in bettering the way both psychiatric disease, and hair loss, are managed.

## Graphical abstract

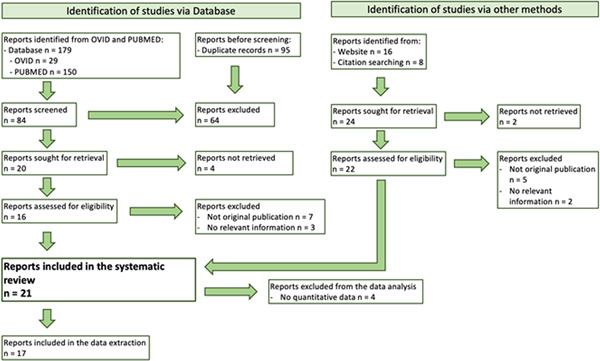

For many years, clinical observations have suggested that there is an intrinsic connection between psychological state and hair disorders. Here, we conducted a systematic review of the literature to assess the relationship between mental health and hair health.

1

**What is already known about this topic?**

- It is generally accepted by dermatologists that there is an association between hair loss and psychological state.
- Alopecia patients who receive psychological support demonstrate improved clinical outcomes.

**What does this study add?**

- We performed a systematic review of the literature and found that the incidence of mental health disorders is significantly higher in patients with alopecia compared to the general population.
- We noted that there were a limited number of studies in this area and concluded that further studies are needed which stratify both the type of alopecia and specific psychiatric disorder.

## 2 INTRODUCTION

It is well accepted by dermatologists, psychiatrists, and their patients that there is an intrinsic connection between psychological state and skin and hair disease.^1^ The relationship between these two health issues is believed to have several layers of interaction.^2^ For example, acute or chronic stress can be the primary inducer of skin diseases such as psoriasis and atopic dermatitis, an aggravating factor for an already existing skin disorder, or a secondary problem in response to skin disease.^2^ While most research to date has focussed on the interconnection between the skin and brain, two recent publications cemented the concept that the nervous system and hair follicle are fundamentally linked. The first of these showed that noradrenaline (a catecholamine) released by the sympathetic nerve in response to acute stress (e.g., nociception‐induced stress) led directly to overstimulation of melanocyte stem cells within the hair follicle and subsequently hair greying.^3^ The second publication showed that corticosterone (cortisol equivalent in rodents; a stress hormone) released by the adrenal gland in response to chronic stress indirectly impacted hair follicle stem cells leading to reduced hair growth.^4^

It is also well accepted that the interaction between the central nervous system and immune cells can be mediated by stress. For example, there are several skin and hair diseases characterized by an inflammatory response, such as atopic dermatitis, psoriasis, and alopecia areata (AA), which are triggered or exacerbated by cognitive dysfunction in the form of stress, depression, and anxiety.^5^ These psychosomatic characteristics are known to modulate neurogenic inflammation and cytokine production, leading to the ‘cytokine hypothesis of depression’.^6^, ^7^ Central to this hypothesis is the idea that psychological stress can cause dysregulated cytokine production, immune cell hyperactivation and hypothalamic‐pituitary‐adrenal (HPA) hyperactivity resulting in depression.^6^ While there is strong evidence linking inflammation and depression, less research has been conducted looking at neuroinflammation in anxiety disorders.^6^

But how do changes in the brain impact the skin and hair? The skin and hair are neuroendocrine organs whose resident cells express stress response neurohormones such as corticotropin‐releasing hormone, adrenocorticotropic hormone and prolactin.^8^ In response to psycho‐emotional stress, these cells express more of these neurohormones.^9^, ^10^ In addition, innervation in the skin, especially surrounding the hair follicle is dense and complex^11^ and consequently acute stress (such as sound stress for 24 h) in mice leads to neuropeptide release from these neurons, otherwise known as neurogenic inflammation.^12^ Neurogenic inflammation is characterized by increased nerve fibre‐mast cell interaction, while increased levels of neuroendocrine stress mediators in the skin leads to degranulation of mast cells and mast cell apoptosis—in turn this degranulation exacerbates skin diseases such as atopic dermatitis.^13^, ^14^ As homoeostasis and the balance between growth and regression states of the hair follicle cycle is intimately connected to the type and number of immune cells in the follicle macroenvironment^13^ this perturbed balance of immune cells in the skin, in response to acute stress, can also manifest as disruptions to the hair cycle. For example, mice exposed to acute stressors have increased perifollicular clustering of macrophages combined with mast cell activation, which leads to an increase in apoptotic cells within the follicle and initiation of follicle regression.^1^

Collectively, the data suggests an important neurogenic and psychological influence on the dynamics of the hair follicle cycle.^11^ However, the brain‐hair follicle axis is two directional and hair loss can be both the cause and consequence of mental health problems.^1^ Psychiatric disorders are more commonly observed in patients with hair loss compared to the general population, with depression, anxiety, somatoform disorders, personality disorders, body dysmorphic disorder, and obsessive‐compulsive disorders (OCD) being the most frequently correlated.^15^ Moreover, hair loss can lead to psychological problems such as low self‐esteem and confidence, introversion, or grief as it is perceived as a failure to conform to society's norms of physical appearance.^15^

So far in this piece, we have used the term hair loss to cover all forms of alopecia, however it is important to note that the way the hair follicle is affected leads to divergent forms of hair loss. There are many forms of hair loss, both scarring and non‐scarring, that affect follicles on the body and scalp, but below we discuss the top four in terms of prevalence. Firstly, androgenetic alopecia (AGA) also known as pattern hair loss, is the most common cause of alopecia in both men and women, affecting 50% of men by the age of 50% and 40% of women by the age of 70.^16^, ^17^ AGA is characterized by a shortened growth phase (anagen) and an elongated resting phase (telogen) of the hair follicle cycle, however the main driving factor in AGA is miniaturization of the follicle and switch from a terminal to a vellus state. This miniaturization does not occur during anagen, but instead occurs during transition through telogen phase of the follicle cycle, meaning increased cycling of the follicle leaves follicles susceptible to miniaturization.^16^ Telogen effluvium (TE) is the second most common form of hair loss after AGA and is characterized by excessive shedding of telogen hair fibres giving the appearance of diffuse hair loss.^16^ Normally hair follicles on the human scalp grow, cycle and shed their fibres in a mosaic pattern, enabling coverage across the scalp at all times.^16^ In TE, which often occurs as a result of stress or trauma, follicles become synchronized and uniformly transition out of anagen, resulting in synchronized entry to telogen and subsequently a synchronized shedding.^16^ On the other hand, anagen effluvium is a non‐scarring diffuse hair loss caused by the impaired mitotic activity of the hair follicle, resulting in a dystrophic anagen hair that sheds. This interruption of hair growth is mostly due to chemo or radiotherapy for treatment of cancer.^18^ Lastly, of relevance for this piece is AA, a non‐scarring, autoimmune‐mediated form of hair loss condition with an incidence of 2% in the general population.^19^ Genetic predisposition, immunological processes, and psychological factors are known to trigger or exacerbate AA, which is caused by a breakdown in immune privilege and cytotoxic T‐cell attack on the hair follicle.^20^ As mentioned above, there are many more forms of hair loss and in the context of this body of work we refer to these collectively as ‘alopecia’.

The way immune cells or perturbations to the follicle cycle can impact the follicle in different ways is abundantly clear. There is also clear evidence that there is an intrinsic hair‐brain axis, so psychological stressors can lead to changes to the hair follicle. What remains uncertain, is if different forms of psychiatric disorder, for example, depression versus anxiety, are characterized by distinct neurogenic inflammation. We hypothesize that different psychiatric disorders are characterized by distinct neurogenic inflammation, which in turn can instigate different forms of hair loss. To find evidence in support of this hypothesis, we systematically collated all publications associated with mental health and hair loss in humans and looked for correlation between specific forms of hair loss and psychological or psychiatric disease.

## 3 METHODS

### 3.1 Search strategy

This systematic review was conducted by searches in April and May 2021 through Ovid MEDLINE and PUBMED using the following protocol. Searches went as far back as 1950 until 2021. The main terms used were alopecia and mental health. Other words were also used to further specify the search such as AA, TE, AGA, female and male pattern hair loss, anagen effluvium, trichotillomania, and tinea capitis. The studies selected for the systematic review were limited to humans. All studies included were English‐language articles focussed on the correlation between alopecia and psychiatry disorders. Moreover, other relevant studies were identified manually through searches in the website Google Scholar, and in the reference lists (referred to as citation searching) of each article found in MEDLINE and PUBMED. In total, 21 articles were selected for inclusion in the systematic review. Of these 17 included quantitative data and were used for data analysis. The study selection was made independently by one of the authors, ALFC.

### 3.2 Study selection

**FIGURE 1:**
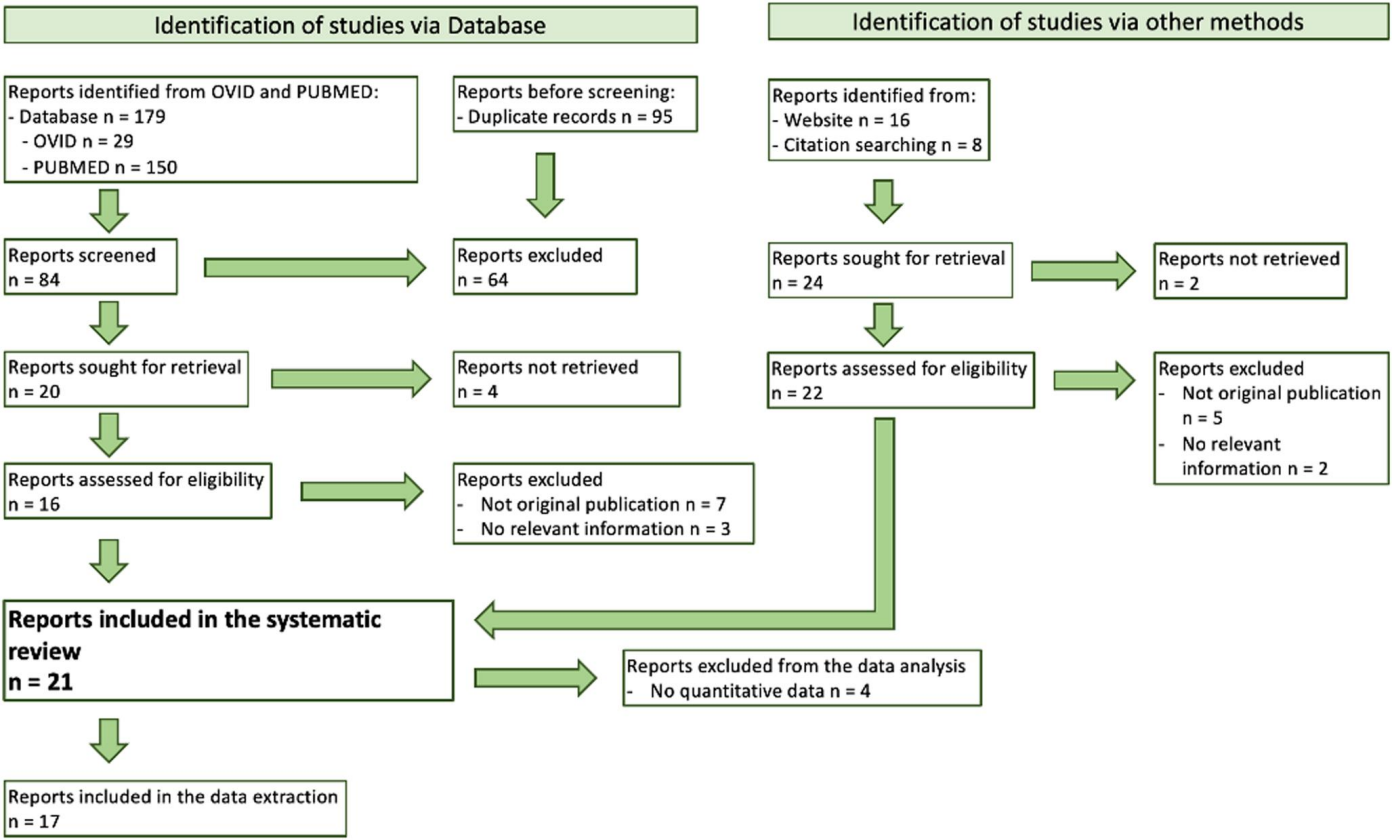
Schematic for the study selection

Studies meeting the following inclusion criteria were selected for full‐text review: (I) the study population contained patients of any age with any hair loss disease that was not induced by drug use; (II) the study reported psychiatric disorders in patients with alopecia using any instrument or questionnaire; and (III) the publications were an original article, a cross‐sectional, clinical, case‐control or report studies. We included studies that reported only scores of any mental health questionnaire in the report results section; however, papers that did not report the prevalence of psychiatric disorders in hair loss patients were not included in the data analysis section. Reporting was in accordance with PRISMA guidelines (Figure 1).

### 3.3 Data extraction and analysis

From these 17 selected articles, patients' data were collected to further analyse the correlation between each hair loss and psychiatry disorder studied. All patient data with absolute numbers, or the percentage for each psychiatric disorder against a total number evaluated, was collected. Only data that specified the number or percentage of patients with a mental health disorder was used, excluding the papers that solely compared the scores of mental health questionnaires. For the statistical analysis of this data, the percentage distribution of patients was analysed with either a *T*‐test (Figure 3a), or a Kruskal‐Wallis test (Figure 5a), on GraphPad Prism 9. Patients from a single publication were regarded as a single datapoint, in the statistical analysis. Statistical analysis was not performed for other figures as there were not enough datapoints to conduct a valid test. This review was not registered.

## 4 RESULTS

### 4.1 Publications collected show alopecia and mental health are causally linked

In total, were able to collect 21 publications about patients with AA, AGA, TE, and other forms of hair loss (termed alopecia), such as lichen planus, frontal fibrosing alopecia, or non‐specified hair loss, who have diagnosed depression, anxiety, have had an acute stress event occur in the past 6 months, or suffer from ‘other’ or non‐specified psychiatric disorders (Table 1). While we specifically searched for patients with anagen effluvium, as this is the third most common form of hair loss, there were no publications linking this form of alopecia with a mental health condition. This is perhaps because anagen effluvium occurs as a side effect to chemotherapy and so this cohort of patients would not be visiting dermatologists for consults for their hair loss condition.

**TABLE 1:** Description of each article identified in this systematic review

Study; location	Study period	Study type	Patient's disease and characteristics	Age	Psychiatric disorder of HrQol
Anderson^21^; United Kingdom	1937–1941	Clinical	114 patients with AA	Not reported	Mental disturbance
Camacho^22^; Spain	1993–1995	Retrospective	‐ 100 female patients with AGA ‐ 100 male patients with AGA	Not reported	MDD and anxiety
Brajac^23^; Croatia	1995–1999	Cross‐sectional	45 patients with AA; ‐ 17 males ‐ 28 females 45 controls; ‐ 22 males ‐ 23 females	20–65 years old	Acute stress
Chu^24^; Taiwan	2000–2009	Case‐control	5117 patients with AA; ‐ 2517 males ‐ 2600 females 20 468 controls; ‐ 10 068 males ‐ 10 400 females	Not reported	MDD, anxiety and obsessive compulsive disorder
Huang^25^; United States	2000–2011	Retrospective cross‐sectional	2115 patients with AA; ‐ 811 males ‐ 1304 females	Mean age 42 years	MDD and anxiety
Güleç^26^; Turkey	2001–2002	Case‐control	52 patients with AA; ‐ 34 males ‐ 18 females	18–65 years	Adjustment disorders
Singam^27^; United States	2002–2012	Retrospective cross‐sectional	5605 patients with AA	Not reported	Several mental health disorders
Ruiz^28^; Spain	2003	Clinical	32 patients with AA ‐ 5 males ‐ 27 females	16–67 years	MDD, anxiety and adjustment disorders
Kivanç^29^; Turkey	2003	Report	248 patients with AGA and TE; ‐ 95 males ‐ 153 females 25 patients with trichodynia ‐ 12 males ‐ 13 females	19–71 years	MDD, anxiety and obsessive personality disorder
Karambetsos^30^; Greece	2004–2009	Cross‐sectional	63 children; ‐ 51 patients ‐ 12 controls ‐ 37 AA	6–14 years	MDD and anxiety
Alfani^31^; Italy	2009–2010	Cross‐sectional	73 patients with AA; ‐ 33 males ‐ 40 females 73 controls; ‐ 33 males ‐ 40 females	Over 18 years	MDD, anxiety, social introversion, obsessiveness and fears
Sellami^32^; Tunisia	2010	Case‐control	50 patients with AA; ‐ 24 males ‐ 26 females	18–60 years	Alexithymia, anxiety and MDD
Schmitt^33^; Brazil	2010	Cross‐sectional	157 females; ‐ 85 with alopecia	Over 21 years	MDD and dysthymia
Ozturk^34^; Turkey	2011	Clinical	31 TE patients with trichodynia 30 TE patients without trichodynia	Over 16 years	Somatoform disorder and anxiety
Tan^35^; China	2012–2013	Clinical	68 patients with AA; ‐ 34 males ‐ 34 females 100 controls	Mean age 34.5 years	MDD, anxiety, obsessive compulsive disorder and phobic anxiety
Tas^36^ ^;^ Turkey	2013–2014	Cross‐sectional	353 patients with AA; ‐ 283 male ‐ 70 female	15–63 years	MDD, anxiety and social phobia
Baghestani^37^; Iran	2015	Case‐control	68 patients with AA 69 controls	Over 18 years	MDD and anxiety
Russo^38^; Italy	2016–2017	Cross‐sectional	143 patients; ‐ 38 males ‐ 105 females 27 AA 80 AGA 36 TE	18–60 years	HrQoL
Montgomery^39^; United Kingdom	2017	Cross‐sectional	338 patients with any type of alopecia (predominantly AA)	13–65 years	MDD, anxiety and social anxiety
Rajoo^40^; Australia	2019	Cross‐sectional	83 patients with AA	Mean age of 41 years	MDD and anxiety
Titeca^41^; Europe	2019	Cross‐sectional multicentre	115 patients with alopecia; ‐ 37 AA ‐ 20 AGA ‐ 58 other alopecia types	Not reported	MDD and HrQoL

Abbreviations: AA, alopecia areata; AGA, androgenetic alopecia; HrQoL, Health‐related Quality of Life; MDD, major depressive disorder; TE, telogen effluvium

#### 4.1.1 Alopecia areata

In total, 16 of the 21 papers included data correlating AA with at least one psychiatric disorder. Several studies demonstrated that AA patients had significantly higher levels of anxiety, depression, stressful life events, and social anxiety^21^, ^23^, ^24^, ^25^, ^26^, ^27^, ^28^, ^30^, ^31^, ^32^, ^33^, ^35^, ^37^, ^39^, ^40^, ^41^ compared to patients without hair loss. Sellami's case‐control study reported that 62% of AA patients have anxiety while 38% have depression.^32^ Chu and colleagues showed that AA patients, regardless of age, are more prone to have anxiety, followed by major depressive disorder (MDD), as appose to any other psychiatry disease.^24^ In contrast, another study by Baghestani showed a higher prevalence of depression (56%) than anxiety (47%) in AA patients.^40^ Chu also showed that schizophrenia was less frequent in AA patients than in the general population^24^ and reported that 50% of the patients developed psychiatric disorders after AA diagnosis while 50% of patients already had a psychiatric disorder, such as depression and anxiety, before the development of AA.^24^ Anxiety, MDD, and OCD preceded AA in 46%, 54%, and 38% of patients, respectively.^24^ In addition, Anderson's paper showed that 23% of patients with AA had a stressful life event preceding the alopecia.^21^

In the paediatric population, Karambetsos et al. demonstrated that AA patients and their parents showed more behaviour alterations, depression, and anxiety than the general population.^30^ In this study, symptoms of anxiety, depression, attention problems, aggressive behaviour, and social problems were more prevalent in the AA group than the control.^30^ Likewise, Liakopoulou et al. reported that all children with AA had anxiety and depression symptoms.^30^ Ghanizadeh also showed that 78% of children with AA had depression.^25^

#### 4.1.2 Androgenetic alopecia

Of the 21 papers identified in the systematic review, five studied the correlation between AGA and psychiatric disorders. These studies associated AGA with anxiety, depression, and other psychiatric disorders such as social phobia and OCD.^22^, ^34^, ^36^, ^38^, ^41^ Interestingly, there was no report of AGA correlated with acute stress. Russo and colleagues demonstrated that females AGA patients are prone to have higher anxiety traits and social phobia than male AGA patients^22^, ^38^ while Camacho's study showed a pattern that women with AGA were more frequently depressed while men with AGA were more anxious and aggressive.^22^ Moreover, AGA patients with trichodynia, a burning and painful sensation in the scalp, were more associated with obsessive personality disorder, low self‐esteem, social phobia, and feelings of social inadequacy compared to the general population.^34^, ^36^

#### 4.1.3 Telogen effluvium

In the 21 papers revised herein, only three articles studied TE and psychiatric disorders. These three papers demonstrated that anxiety and other psychiatric disorders are correlated with TE, while there was no report of depression and acute stress in these patients.^29^, ^34^, ^38^ Trichodynia, usually related to TE, was additionally correlated to depression, anxiety disorder, and obsessive personality disorder.^34^ Kivanç et al. showed that 76.5% of patients with TE associated with trichodynia were diagnosed with depression.^29^

#### 4.1.4 Alopecia

In total, three articles reported the correlation between hair loss and psychiatric disorders without specifying the type of alopecia. Depression and other psychiatric disorders were more prevalent in alopecia patients than anxiety and acute stress.^33^, ^39^, ^40^ Schmitt and colleagues' study demonstrated a significant association between hair loss complaints and depressive symptoms.^33^ The study reported that 38% of hair loss patients presented major depressive symptoms, such as anhedonia and sadness.^33^, ^40^

### 4.2 Over two thirds of publications were focussed on AA

**FIGURE 2:**
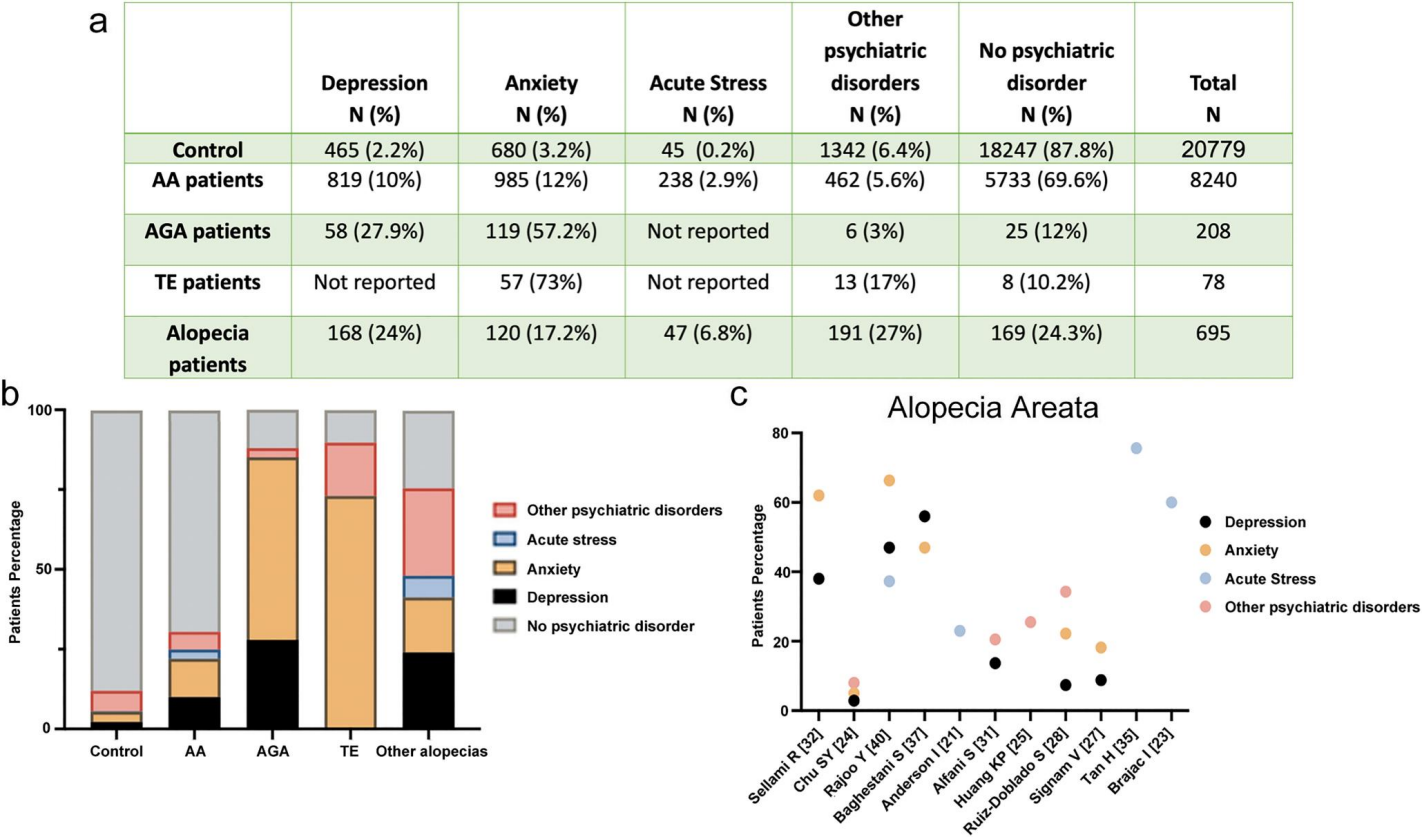
(a) Patients' data collected from the analysed publications. (b) Distribution of psychiatric disorders in each hair loss disease based on data in (a). (c) Heterogeneity of psychiatric disorder distribution in 11 publications with quantitative data in AA. AA, alopecia areata.

In total, from the 21 publications identified, only 17 specified the number or percentage of patients with mental health disorders (Table 2). Publications by Karambetsos,^30^ Güleç,^26^ Russo,^38^ and Tas^36^ were therefore excluded from the data analysis since they do not report quantitative data. In total, there were 20 779 controls and 9221 patients; 8240 with AA, 208 with AGA, 78 with TE, and, lastly, 695 with other forms of alopecia, such as lichen planus and frontal fibrosing alopecia, or forms that were not specified in the papers (Figure 2a). When patients had more than one psychiatric condition, for example, depression and anxiety, they were classified as having ‘other psychiatric disorders’, however this category also includes other psychiatric disorders such as OCD or body dysmorphic disorder. Moreover, six articles analysed used the general healthy population as a control group to compare the prevalence of psychiatric disorders to the patient group under analysis, and so we incorporated this data into our study as a control dataset. Immediately striking from this data is that despite being the fourth most common form of hair loss, the number of patients with AA seeking consult is considerable higher than for other forms of hair loss. Collectively, when the patients are compared together, we can see that alopecia types are more associated with psychiatric disorders than the general population (Figure 2b). However, in contrast to common belief, it appears from the outset that AA patients do not have a higher prevalence of psychiatric disorders than other hair loss diseases. In fact, higher levels of psychiatric disorders are observed in TE and AGA patients. This data is however likely to be biased as we collectively assessed the total number of patients together, with AA patients accounting for almost 90% of those analysed. We therefore conducted further analysis by looking at the prevalence of psychiatric disorders within each of the 17 manuscripts with quantitative data and considered each of these as an individual dataset.

**TABLE 2:** Distribution of psychiatric disorder prevalence in hair loss patients in each publication

Anderson^21^	Alopecia areata	Anxiety: 23%
Camacho^22^	Androgenetic alopecia	Depression: 29%
		Anxiety: 59.5%
Kivanç^29^	Androgenetic alopecia	Other psychiatric disorders: 75%
Kivanç^29^	Telogen effluvium	Other psychiatric disorders: 76.4%
Ruiz^28^	Alopecia areata	Depression: 7.4%
		Anxiety: 22.2%
		Other psychiatric disorders: 34.4%
Brajac^23^	Alopecia areata	Acute stress: 60%
Chu^24^	Alopecia areata	Depression: 2.9%
		Anxiety: 5%
		Other psychiatric disorders: 8%
Schmitt^33^	Alopecia	Depression: 29%
		Anxiety: 19.7%
Alfani^31^	Alopecia areata	Depression: 13.7%
		Anxiety: 13.7%
		Other psychiatric disorders: 20.5%
Ozturk^34^	Telogen effluvium	Anxiety: 93.4%
Huang^25^	Alopecia areata	Other psychiatric disorders: 25.5%
Sellami^32^	Alopecia areata	Depression: 38%
		Anxiety: 62%
Baghestani^37^	Alopecia areata	Depression: 56%
		Anxiety: 47%
Tan^35^	Alopecia	Acute stress: 75.6%
Montgomery^39^	Alopecia	Depression: 29%
		Anxiety: 35.5%
		Other psychiatric disorders: 47.5%
Rajoo^40^	Alopecia areata	Depression: 47%
		Anxiety: 66.3%
		Acute stress: 37.3%
Singam^27^	Alopecia areata	Depression: 8.8%
		Anxiety: 18.2%
Titeca^41^	Alopecia	Acute stress: 40.8%

Since AA was the hair loss type with the largest number of publications (16 papers out of 21 articles reviewed and 11 with quantitative data), we plotted the distribution of the percentage of AA patients with psychiatric disorders (Figure 2c). This data showed that depression and anxiety are the most studied psychiatric disorder in AA patients within the seven of the 11 articles with quantitative data including these disorders.

### 4.3 Psychiatric disease is significantly more prevalent in patients with hair loss

**FIGURE 3:**
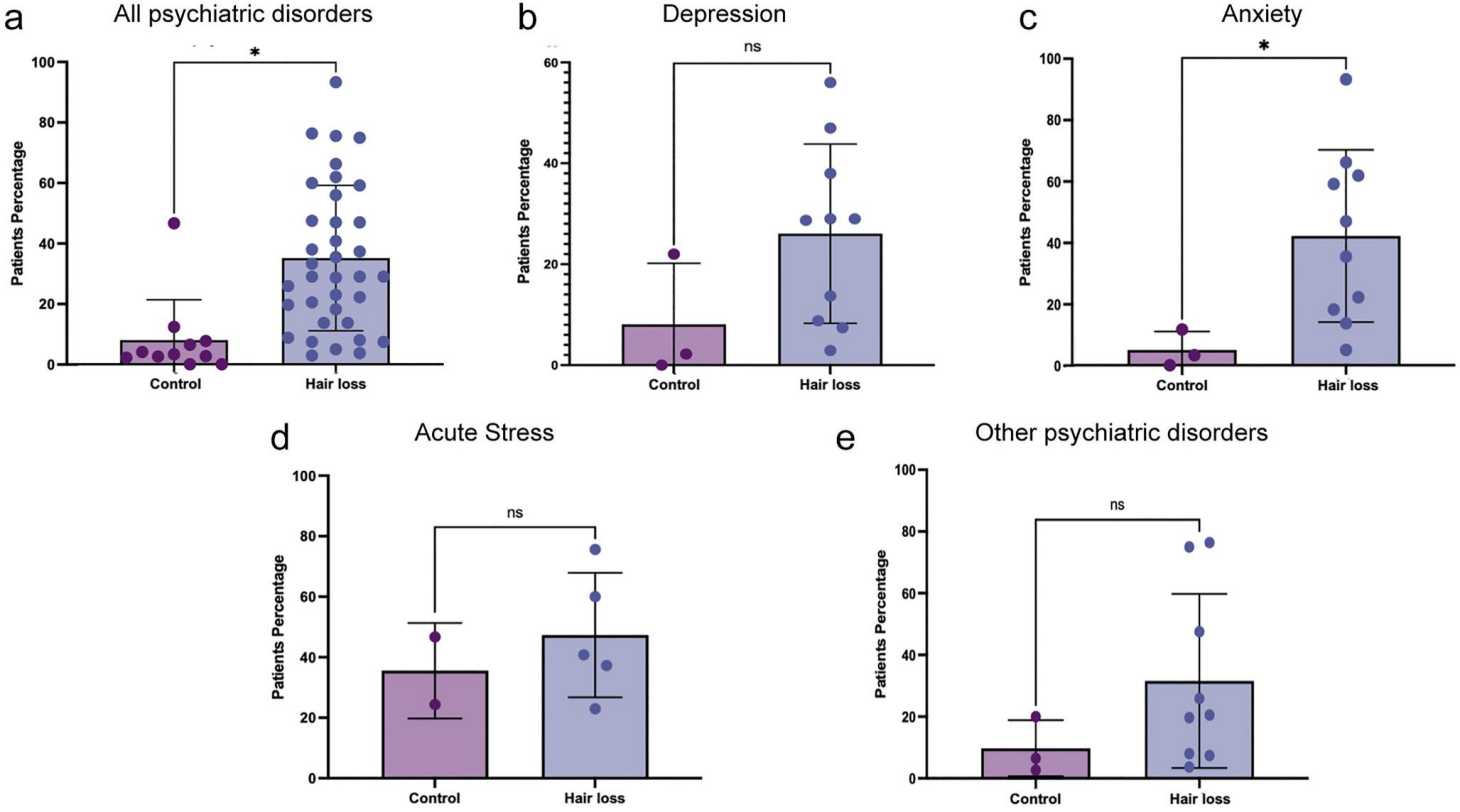
(a) The broad correlation between all hair loss and any psychiatric disorder shows 35.1% of hair loss patients with psychiatric disease compared to 8% in the general population (*p* = 0.0009). (b) The correlation between all hair loss and depression (*p* = 0.1337), (c) between hair loss and anxiety (*p* = 0.0490), (d) between hair loss and acute stress events (*p* = 0.5596) and lastly (e) between hair loss and other psychiatric disorders reveals in all cases that psychiatric disorders tend to be more prevalent amongst patients with hair loss compared to those without (*p* = 0.2288).

Initially, to address the age‐old question of whether psychological disorders are more prevalent in patients with hair loss or alopecia, we collectively compared the prevalence of all psychological disorder within the general population and all hair loss patients (AA, AGA, TE, and other alopecia patients together) from the 17 manuscripts with quantitative data identified in the systematic review. We found that patients with hair loss have significantly more mental health disorders than the control group (*p* = 0.0009) (Figure 3a); approximately 8% of control patients have a mental health disorder compared to 35.1% of patients with hair loss. Therefore, the broad correlation well accepted to patients and physicians is demonstrated to be accurate herein. To further study this broad correlation, we then looked at each psychiatric disorder; depression, anxiety, acute stress or other, in turn, and compared prevalence in the general population against all hair loss patients. Analysing the prevalence of depression of the general population and AA, AGA, TE, and alopecia patients altogether, it is possible to observe that hair loss patients have more depression than the control group (26% vs. 8%) (*p* = 0.1337) (Figure 3b), more anxiety than the general population (42.2% vs. 3.3%) (*p* = 0.0490) (Figure 3c) while 45% of the patient group had been subject to stressful life events in the prior 6 months compared to 35.5% in the control group (*p* = 0.5596) (Figure 3d). Lastly, comparing the prevalence of other psychological disorders, such as OCD and social phobia, to the general population and all hair loss patients, we again found a higher incidence in all patients with hair loss compared to the control population (31.6% vs. 6.5%) (*p* = 0.2288) (Figure 3e).

**FIGURE 4:**
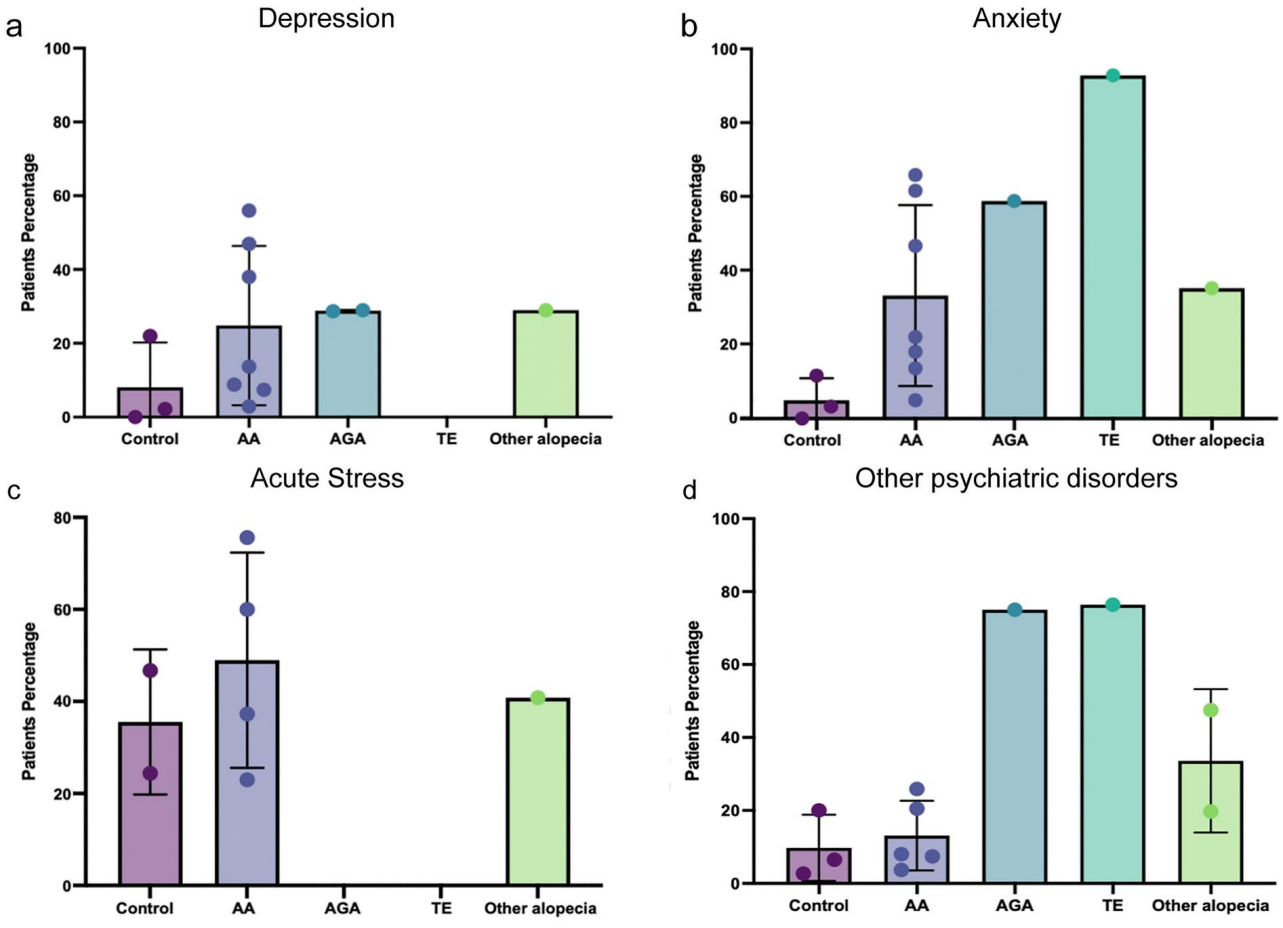
(a) Depression prevalence in each hair loss disease shows relatively similar incidence to the control, however this is higher than WHO estimate, which puts depression at 4.5% in healthy individuals. There were no publications associating TE with depression. (b) Anxiety prevalence distribution in hair loss patients and in the general population shows higher levels of anxiety in all hair loss types relative to levels in the general population. (c) The correlation between acute stress and hair loss was only reported in AA and other alopecia patients, not in AGA or cases of TE. (d) The association of several other psychiatric disorders with each hair loss disease suggests highest prevalence in AGA and TE, however in each case this is a single datapoint from one publication. AA, alopecia areata; AGA, androgenetic alopecia; TE, telogen effluvium.

Next, to see if specific mental health disorders were correlated with different forms of hair loss, we compared across types of hair loss (as appose to treating them as a single condition) for the incidence of specific psychological disease, for example, depression or anxiety. First, looking at depression, we found prevalence in patients with all forms of hair loss (with the exception of TE where there was no associated publication), to be slightly higher than the general population (Figure 4a). The general population in our study though had a depression prevalence of 8%, while World Health Organisation data suggests levels are around 4.5% in adults.^42^ This would suggest that hair loss is associated with an increased incidence of depression. When we looked at anxiety, patients with all types of hair loss showed a higher prevalence of anxiety than the general population. TE patients were found to the most anxious, compared to AGA, AA, and others alopecia patients, however this TE datapoint was collected from just a single publication (Figure 4b). In the acute stress analysis, stressful life events were not reported in either AGA and TE patients. Even though there is little data about acute stress events (just four publications), we saw that both AA patients and other alopecia patients had on average been exposed to more acute stress events in the prior 6 months compared to the general population (Figure 4c). Lastly, when we assessed other psychiatric disorders, which includes social phobia, OCD, or multiple psychiatric conditions combined (for example depression and anxiety), we observed a higher incidence in AGA, TE, and other alopecia patients, compared to the control group (Figure 4d). It is worth noting that there were only two publications; one for AGA, and one for TE, which grouped all psychiatric conditions together within their manuscripts. The manuscripts were included in our analysis, and are hence presented in graphical form, however this clustering of any condition under the banner of other psychiatric disorders means there is perhaps under‐representation of depression in TE or AGA patients and over‐representation of other psychiatric disorders.

**FIGURE 5:**
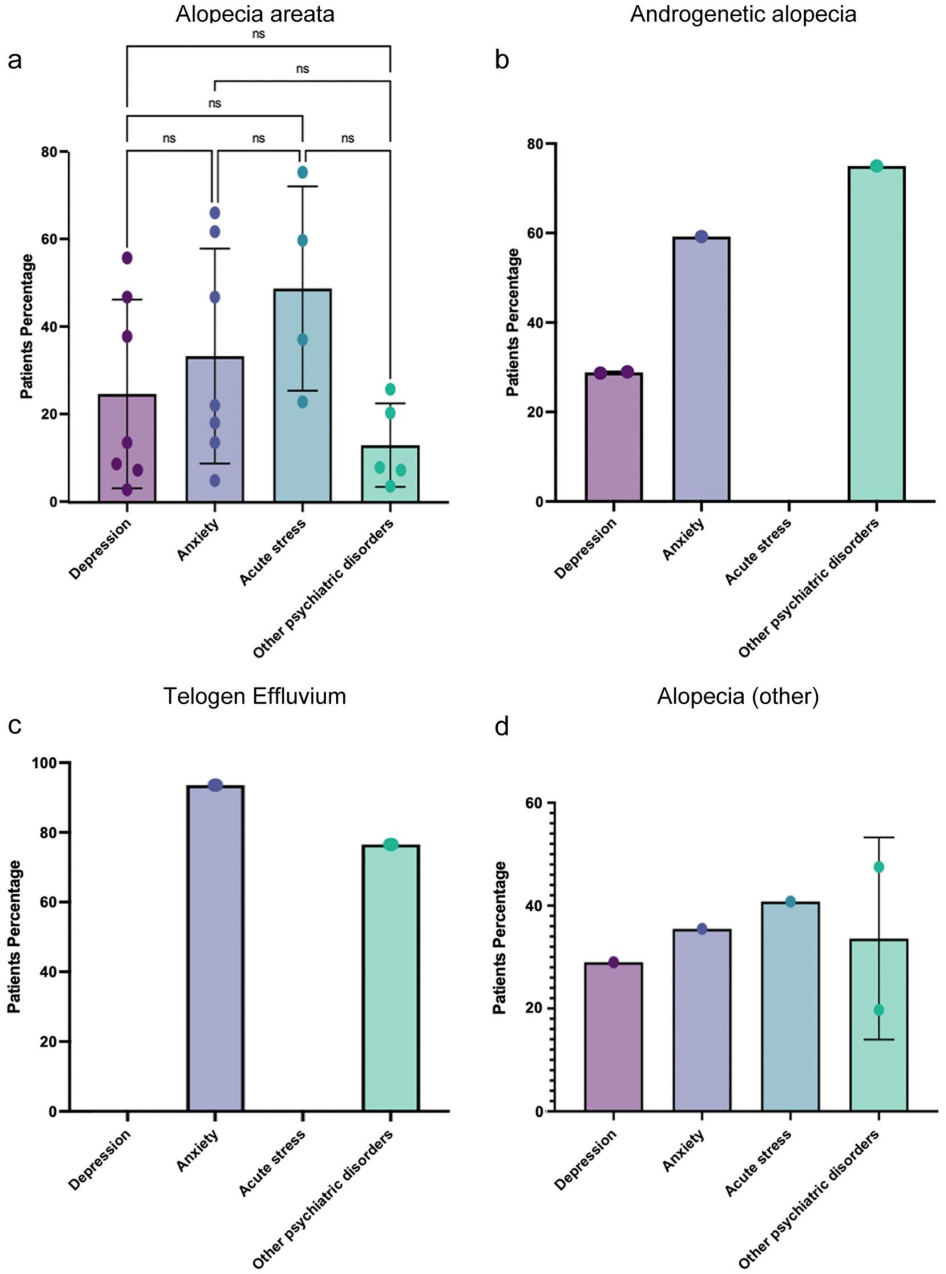
(a) Distribution of the psychiatric disorders in AA patients shows highest levels of acute stress followed by anxiety, depression, and other psychiatric disorders; however, acute stress was not significantly more prevalent compared to other disorders. (b) The correlation between each psychiatric disorder in AGA patients indicated high levels of other psychiatric disorders although this data was from a single publication. (c) In TE patients there were just two publications, one linking TE with anxiety and the other linking it with other psychiatric disorders. (d) In non‐specified alopecia all types of psychiatric disorders showed high prevalence but there was not a large distinction between diagnoses. AA, alopecia areata; AGA, androgenetic alopecia; TE, telogen effluvium.

We next evaluated the data to see if there was a correlation between specific forms of hair loss and a specific psychological disease. First, we analysed the prevalence of each psychiatric disorder in AA patients. We found that acute stress events are the most prevalent mental health disorder in AA patients (48.9%), followed by anxiety (33.5%), depression (24.8%), and other psychiatric disorders (13.1%) (*p* = 0.1251) (Figure 5a). When comparing the prevalence of each psychiatric disorder in AGA patients, we found that 75% of patients had other psychiatric disorders, 59.2% had anxiety, and 28.8% had depression. It is important to note that there were no reports of acute stress in these patients (Figure 5b). TE had only two studies reporting the number or percentage of patients; one focussed on anxiety indicating prevalence of 93.4% while the second publication reported other psychiatric disorders at a prevalence of 76.4% (Figure 5c). Lastly, the prevalence of each psychiatric disorder was compared in alopecia patients. In this analysis, we found that acute stress was the most prevalent mental health disorder in other alopecia patients (40.8%), followed by anxiety (35.5%), acute stress (33.6%), and depression (29.0%) (Figure 5d).

## 5 DISCUSSION

Stress is a broad term that can include multiple physical or emotional strains in a person. For this reason, from the psychoneuroimmunology perspective, psychiatric disorders can be interpreted as stressors.^11^ Stress can induce or exacerbate mental health disorders,^43^ but where does hair loss come in? The nervous system, the skin and hair follicle have an intimate connection and interaction throughout life since these two systems have the same embryonic origin, the ectoderm. Studies have shown that around 50% of patients with alopecia suffer from a psychological problem associated with their hair loss. Moreover, studies have demonstrated that many patients receiving psychological support during their hair loss treatment have improved disease prognosis.^37^ However, there is a significant limitation in terms of research, especially since the association between alopecia and depression may be confounded with stressful life events, which trigger both disorders.^14^

In this systematic review, we identified and assessed 21 publications (17 with quantitative data) from a starting pool of 201, to try and determine if there is a correlation between specific forms of hair loss and specific types of psychological disease. A two‐way correlation is very perceptible in patients with skin diseases, for example, psoriasis and atopic dermatitis are known to have a cause and consequence relationship with depression and anxiety. In the same way that acute or chronic mood disorders induce or worsen skin conditions, skin diseases also cause psychological impairments and disorders in around 30% of patients.^44^ We hypothesize that the manifestation of different types of psychological disease, and the impact of this on the brain and later the skin, will also lead to variations in hair follicle response, initiating different forms of hair loss.

The correlation between psychological problems and hair loss seems to be very well consolidated by physicians and patients, even though there are limited studies on this topic. In our study, we were able to observe trends of the more specific correlation between each hair loss type and each psychiatric disorder. Acute stress seems to be more associated with AA and alopecia, while anxiety is more correlated with TE. In addition, AGA patients show most association with other psychiatric disorders, such as social phobia and OCD. Despite the low number of publications identified, it is undebatable that hair loss greatly affects Health‐related Quality of life (HrQol) scores and it is strongly associated with mental health disorders, especially depression and anxiety. Increased knowledge about this association is needed to better treat these patients. For this reason, hair loss patients may have a better prognosis from the use of antidepressants, anxiolytics, or even psychotherapy.^24^, ^41^ Therefore, more extensive studies, and coordination or collaboration between psychiatrists and dermatologists could help to improve the outcome of both mental health disorders and hair loss.

## CONFLICT OF INTEREST

None to declare.

## AUTHOR CONTRIBUTIONS

**Ana Forneris‐Crego:** Conceptualization (Equal); Data curation (Lead); Formal analysis (Lead); Investigation (Lead); Methodology (Equal); Visualization (Lead); Writing – original draft (Lead); Writing – review & editing (Equal). **Anastasia Therianou:** Conceptualization (Supporting); Writing – review & editing (Supporting). **Parastoo Hashemi:** Formal analysis (Supporting); Writing – review & editing (Supporting); **Claire Higgins:** Conceptualization (Equal); Investigation (Supporting); Methodology (Lead); Writing – original draft (Lead); Writing – review & editing (Lead).

## ETHICS STATEMENT

Not applicable.

## Data Availability

No data are available.
